# Casson nanoliquid film flow over an unsteady moving surface with time-varying stretching velocity

**DOI:** 10.1038/s41598-023-30886-4

**Published:** 2023-03-11

**Authors:** G. P. Vanitha, K. C. Shobha, B. Patil Mallikarjun, U. S. Mahabaleshwar, Gabriella Bognár

**Affiliations:** 1 Department of Mathematics, Siddaganga Institution of Technology, Tumkur, Karnataka India; 2 Department of Studies and Research in Mathematics, Tumkur University, Tumakuru, Karnataka 572103 India; 3 Department of Mathematics, Davanagere University, Davanagere, Karnataka India; 4 Institute of Machine and Product Design, University of Miskolc, Miskolc, 3515 Egyetemváros Hungary

**Keywords:** Mechanical engineering, Applied mathematics

## Abstract

Present study explains about unsteady Casson nanoliquid film flow over a surface moving with velocity $U_w=\lambda x/t$. The governing momentum equation is reduced to ODE by using corresponding similarity transformation, which is then tackled by employing numerical technique. The problem is analysed for both two-dimensional film flow and axisymmetric film flow. The exact solution is derived which satisfies the governing equation. It is noted that solution exists only for a specified scale of the moving surface parameter $\lambda$. ie., $\lambda \ge -1/2$ for two-dimensional flow and $\lambda \le -1/4$ for axisymmetric flow. The velocity increases first and reaches the maximum velocity and then decreases to the boundary condition. Streamlines are also analysed for both axisymmetric and two-dimensional flow patterns by considering the stretching ($\lambda >0$) and shrinking wall conditions ($\lambda <0$). Study has been made for large values of wall moving parameter $\lambda$. The aim of this investigation is to analyse the Casson nanoliquid film flow which finds applications in industries like coating of sheet or wire, laboratories, painting, many more.

## Introduction

For the purpose of comprehending and designing various heat exchangers and industrial processing machinery, it is crucial to understand flow and heat transmission inside a thin liquid layer. Wire and fibre coating, polymer processing, reactor fluidization, evaporation cooling, food processing and other common uses are a few applications. The manufacturing of polymeric sheets, paper, linoleum, insulator components, roofing shingles, fine fibre mattes, boundary layers along liquid films in condensation techniques, etc. need the thermal processing of sheet-like components^1^. Often, the sheet moves along its own plane throughout such processing procedures. The fluid beside the moving sheet may move independently of it or alternately, the fluid may move parallel to the sheet’s motion due to forced convection. A high-quality extrudate surface is the goal of every extrusion procedure. For better product quality, it is crucial to regulate the drag and energy flux. Owing to the enormous capacity of nanofluids to be employed as technical instruments in several engineering fields, an increasing number of researchers are now paying attention to examine the laminar flow of a thin liquid layer across stretching sheet. In view of all the above applications, Sparrow and Gregg^2^ initially investigated the problem of laminar-film condensation on a vertical plate by employing the theory of the boundary layer flow and similarity transformations. Then they extended the work to analyse the heat and mass transmission in a liquid film on a spinning disc^3^. Wang^4^ examined the melting from a horizontally spinning disc and the nonlinear system of equations were tackled by employing the perturbation technique. Dandapat and Ray^5,6^ enquired the thermal capillarity and cooling impacts on a thin liquid sheet over a revolving disc. Wang^7^ was the first to take into account the hydrodynamics of a thin liquid layer on a stretching sheet after employing similarity transformation to convert the unstable Navier–Stokes equations into nonlinear ordinary differential equations. With review of different velocity and thermal boundary conditions, Wang’s work was further developed by other researchers, including Usha and Sridharan^8^, Chen^9,10^, Andersson et al.^11^, Abbas et al.^12^, Liu and Andersson^13,14^, Wang^15^ and Dandapat et al.^16–18^. Mahabaleshwar et al.^19,20^ analysed an electrically conducting Newtonian fluid flowing past a superlinear stretching/shrinking sheet with MHD. Jitendra et al.^21^ scrutinized the nonlinear hydrodynamical magnetic boundary layer laminar flow of a viscous, incompressible fluid past a porous stretching sheet with suction/injection.

The scope of the aforementioned studies was solely the laminar flow of Newtonian (pure) fluids. However, in recent times, the science and engineering community has been interested in nanofluids (a term coined by Choi^22^) because of their industrial uses. Because of the distinct physical and chemical characteristics that nanoscale materials have, nanotechnology is a new discipline with widespread application in business. Colloidal suspensions of various materials, oxide, metallic and non-metallic, silicon carbide, or carbon nanotubes in a base fluid are present in these fluids. Ethylene glycol and water are typical base fluids. Numerous heat transfer processes, such as those in fuel cells, microelectronics, pharmaceutical manufacturing, and hybrid power engines, may benefit from the features of nanofluids. Comparing to the base fluids, they exhibits improved heat transfer coefficients and thermal conductivities. Because of this, nanofluids are frequently preferred to traditional coolants such oils made of water and ethylene glycol. The latest work by Das et al.^23^ and review articles by Ding et al.^24^, Buongiorno^25^, Wang and Mujumdar^26,27^, Daungthongsuk and Wongwises^28^, Kakaç and Pramuanjaroenkij^29^ provide thorough references on nanofluid. Greater thermal conductivity in thermal systems can be achieved using nanofluids, see Eastman et al.^30^ and Xie et al.^31^. Kumari and Nath^32^ investigated the unsteady MHD film on a continuously rotating disc. Mahabaleshwar^33^ considered linear stretching sheet problem in porous domain with suction. Recent works on the flow of hybrid nanofluids over differenr geometries has been repoted in^34–45^.

The goal of this research is to apply the mathematical nanofluid model to analyse the unstable casson nano-liquid film formed by a linear stretching velocity across a moving surface. In this paper, we consider the wall velocity as $U_w=\lambda x/t$. The model is expressed, analyzed and numerically solved in the parts that follow. The most important findings are graphically presented and explained for both axisymmetric and two dimensional cases.

## Mathematical formulation and solution

**Figure 1:**
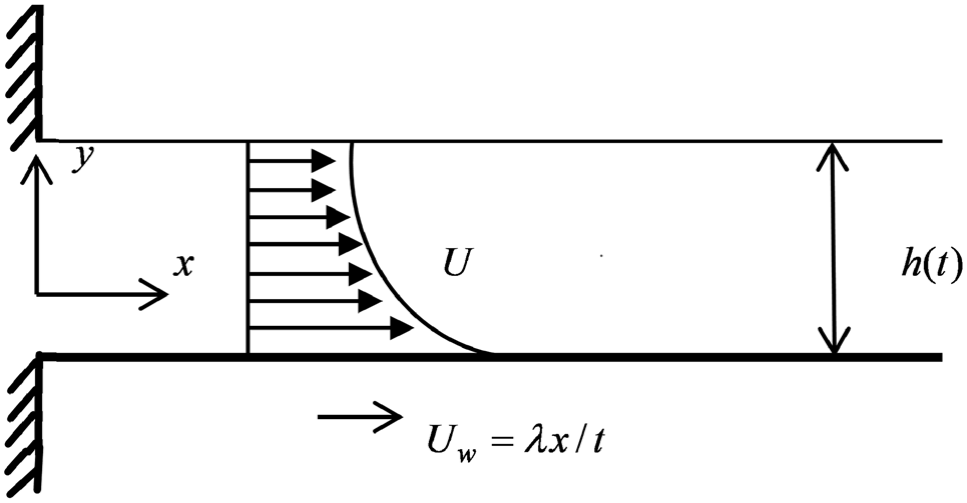
Schematic representation of the problem.

Let us consider the incompressible, laminar flow of Casson nanofluid over an unsteadily moving flat sheet moving with velocity $u_w(x, 0, t)$. The fluid film is presumed to be steady and have a fixed thickness *h*(*t*) on the surface and remains flat at all the time. Assuming the ambient gas pressure to be $p_0$, film is considered as free surface with ambient gas at the interface. The wall is taken to be permeable, which gives $v_w(x,0, t)=v_w$. Cartesian co-ordinate system is chosen so that x-axis is considered to be along the plate and y-axis is orthogonal to it as depicted in Fig. 1. The governing Navier–Stokes equations^46^ of this considered problem takes the form

1 $$\begin{aligned}{} & {} \frac{\partial u}{\partial x}+\epsilon \frac{u}{x}+\frac{\partial v}{\partial y}=0, \end{aligned}$$

2 $$\begin{aligned}{} & {} \frac{\partial u}{\partial t}+u \frac{\partial u}{\partial x}+v \frac{\partial u}{\partial y} = - \frac{1}{\rho _{nf}} \frac{\partial p}{\partial x}+\nu _{nf} \left[ \frac{\partial ^2 u}{\partial x^2}+\frac{\partial ^2 u}{\partial y^2}\left( 1+\frac{1}{\Gamma }\right) + \nu _{nf} \epsilon \left( \frac{1}{x}\frac{\partial u}{\partial x}-\frac{u}{x^2}\right) \right] , \end{aligned}$$

3 $$\begin{aligned}{} & {} \frac{\partial v}{\partial t}+u \frac{\partial v}{\partial x}+v \frac{\partial v}{\partial y} = - \frac{1}{\rho _{nf}} \frac{\partial p}{\partial y}+\nu _{nf} \left[ \frac{\partial ^2 v}{\partial x^2}+\frac{\partial ^2 v}{\partial y^2}\left( 1+\frac{1}{\Gamma }\right) + \nu _{nf} \epsilon \left( \frac{1}{x}\frac{\partial v}{\partial x}\right) \right] , \end{aligned}$$

exposed to the constraints

4 $$\begin{aligned}{} & {} u(x, 0, t) = U_w,\nonumber \\{} & {} v(x, 0, t) = v_w,\nonumber \\{} & {} \frac{\partial u}{\partial y}(x, h, t) = 0, \end{aligned}$$

where *u* and *v* specifies the velocity components in the *x* and *y* directions, $\nu$ denotes the kinematic viscosity, $\rho$ denotes the density, *p* denotes the fluid pressure, $B_0$ specifies the applied magnetic field strength applied with an angle of inclination $\alpha$. $\epsilon =0$ specifies the 2-dimensional flow and $\epsilon =1$ specifies the axisymmetric flow pattern in the governing equations. Let us consider the velocity of the unsteadily moving wall as $U_w=\frac{\lambda }{t}x$ where $\lambda$ is a real number. As there is no pressure difference in the *x* direction along the interface, pressure field depends only on *y* and *t* for the considered flow configuration. The governing Eqs. (1) to (3) can be modified to similarity equations by defining the stream function and similarity variable^46^ as:

$$\begin{aligned} \psi (x, y, t)=\sqrt{\frac{\nu }{t}}x^{1+\epsilon }f(\eta ) \end{aligned}$$

and

5 $$\begin{aligned} \eta = \frac{y}{\sqrt{\nu t}}. \end{aligned}$$

Then the components of velocity takes the form $u=\frac{1}{x^\epsilon }\frac{\partial \psi }{\partial y}=\frac{x f''(\eta )}{t}$ and $v=-\frac{1}{x^\epsilon }\frac{\partial \psi }{\partial x}=-(1+\epsilon )\sqrt{\frac{\nu }{t}}f(\eta )$. Expression for pressure field can be reduced to:

6 $$\begin{aligned} -p(x, y, t)=\rho _{nf} (1+\epsilon )\frac{\nu }{t}\left[ f'(\eta )+\frac{(1+\epsilon )f^2(\eta )}{2}-\frac{f(\eta )\eta }{2}\right] +C(t) \end{aligned}$$

The effective viscosity and density of nanofluids can be expressed as^47^

7 $$\begin{aligned} \mu _{nf}=\frac{\mu _{f}}{(1-\phi )^{2.5}},\nonumber \\ \rho _{nf}=(1-\phi )\rho _{f}+\phi \rho _{s}. \end{aligned}$$

Using these, the governing equations reduces as

8 $$\begin{aligned} A\left( 1+\frac{1}{\Gamma }\right) f'''+(1+\epsilon ) f f''-f'^2+\left[ \frac{\eta }{2}f''+f'\right] = 0, \end{aligned}$$

where, $A=\frac{1}{(1-\phi )^{2.5}\left[ 1-\phi +\phi \frac{\rho _{s}}{\rho _{f}}\right] }$ and the corresponding boundary constraints become

9 $$\begin{aligned} f(0)=0, \quad f'(0)=\lambda \quad \text {and} \quad f''(\beta )=0, \end{aligned}$$

where $\beta$ denotes the non-dimensional thickness of the film, $\lambda$ is known as the wall moving parameter in which $\lambda >0$ designates stretching and $\lambda <0$ designates shrinking. The thickness of the film *h*(*t*) is evidenced as $h(t)=\beta \sqrt{\nu t}$. Comparing the surface vertical velocity $h'(t)=\frac{\beta \sqrt{\nu }}{2\sqrt{t}}=-(1+\epsilon )\sqrt{\frac{\nu }{t}}f(\eta )$ gives one more boundary constraint $f(\beta )=-\frac{\beta }{2(1+\epsilon )}$. Transformation $f(\eta )=\beta F(\frac{\eta }{\beta })=\beta F(\xi )$ is used in order to simplify the calculations, which gives

10 $$\begin{aligned} F_{\xi \xi \xi }+\frac{\beta ^2}{A\left( 1+\frac{1}{\Gamma }\right) }\left[ (1+\epsilon )FF_{\xi \xi }-F_{\xi }^2+\left( \frac{\xi }{2}F_{\xi \xi }+F_{\xi }\right) \right] = 0 \end{aligned}$$

exposed to the constraints

11 $$\begin{aligned} F(0)=0, \quad F_{\xi }(0)=\lambda , \quad F_{\xi \xi }(1)=0 \quad \text {and} \quad F(1)=-\frac{1}{2(1+\epsilon )}. \end{aligned}$$

## Results and discussions

Since we cannot obtain the exact solutions of Eqs. (9) and (10) analytically, it is required to use numerical techniques for the solution of the problem. Here we have used bvp4c MATLAB code which uses the finite difference method procedure to tackle the similarity equations for various flow parameters. The obtained numerical solutions are discussed and analysed in the "Numerical solutions" and "Solution behaviour analysis for large λ" sections.

### Numerical solutions

To analyse the characteristic of the solution behaviour, integrating the similarity equation once, we obtain the expression as:

12 $$\begin{aligned} F_{\xi \xi }+\frac{\beta ^2}{A\left( 1+\frac{1}{\Gamma }\right) }\left[ (1+\epsilon )FF_\xi -(2+\epsilon )\int _{0}^{\xi }F_\xi ^2 d\sigma +\frac{\xi }{2}F_\xi +F\right] = C_1. \end{aligned}$$

Imposing the boundary condition at $\xi =0$ gives $C_1=F''(0)$. Again applying the boundary constraints at $\xi =1$ and reordering the terms gives

13 $$\begin{aligned} F_{\xi \xi }(0)=-\frac{\beta ^2}{A\left( 1+\frac{1}{\Gamma }\right) }\left[ (2+\epsilon )\int _{0}^{1}F_\xi ^2 d\sigma +\frac{1}{2(1+\epsilon )}\right] \end{aligned}$$

Further integrating this expression yields

14 $$\begin{aligned} F_\xi +\frac{\beta ^2}{A\left( 1+\frac{1}{\Gamma }\right) }\left[ \frac{(1+\epsilon )}{2}F^2-(2+\epsilon )\int _{0}^{\xi }\int _{0}^{\tau }F_\xi ^2d\sigma d\tau +\frac{\xi F}{2}-\int _{0}^{\xi }F d\sigma \right] =F_{\xi \xi }(0)\xi +A. \end{aligned}$$

**Figure 2:**
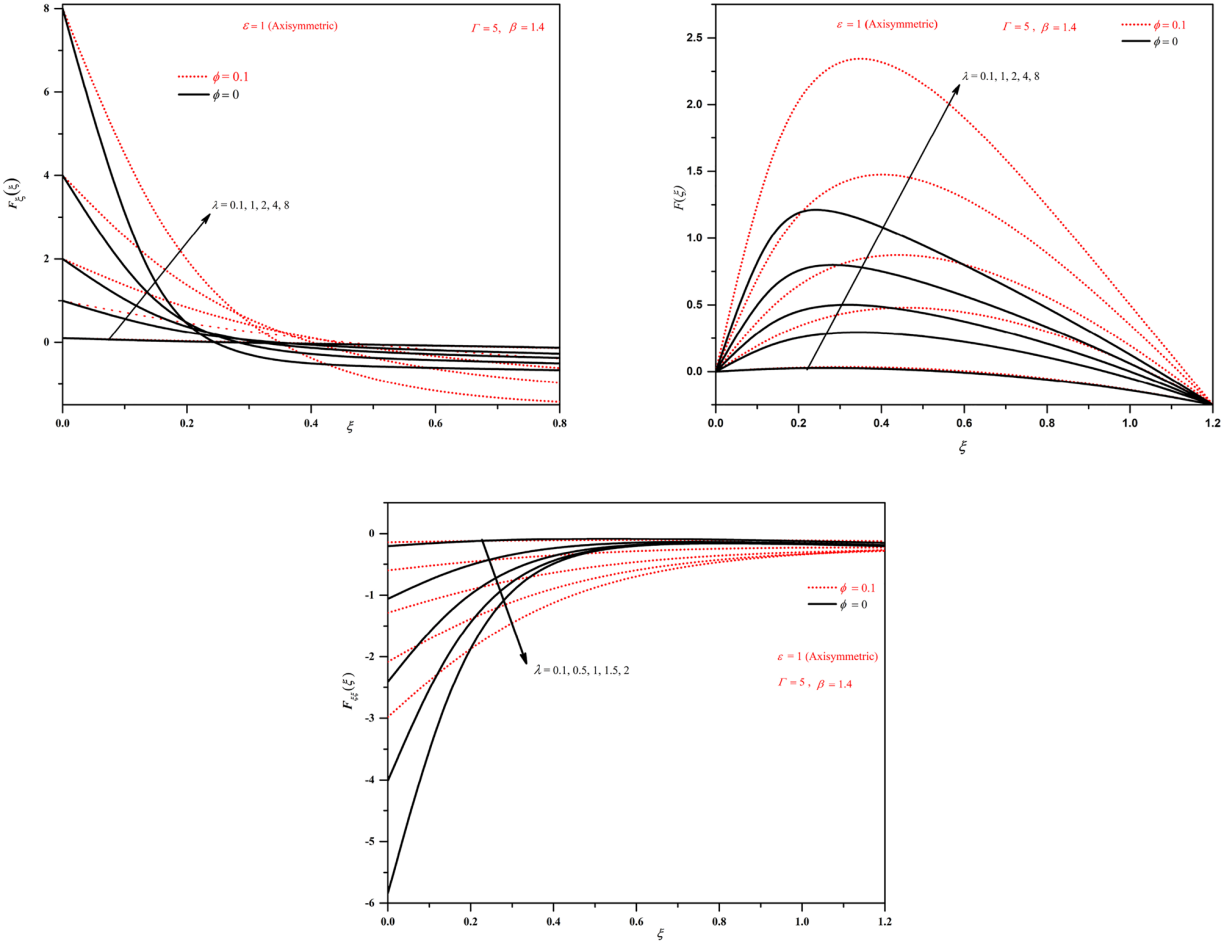
Plots of *F*, $F_{\xi }$ and $F_{\xi \xi }$ by varying $\lambda$ (stretching wall $\lambda >0$) in the axisymmetric case.

**Figure 3:**
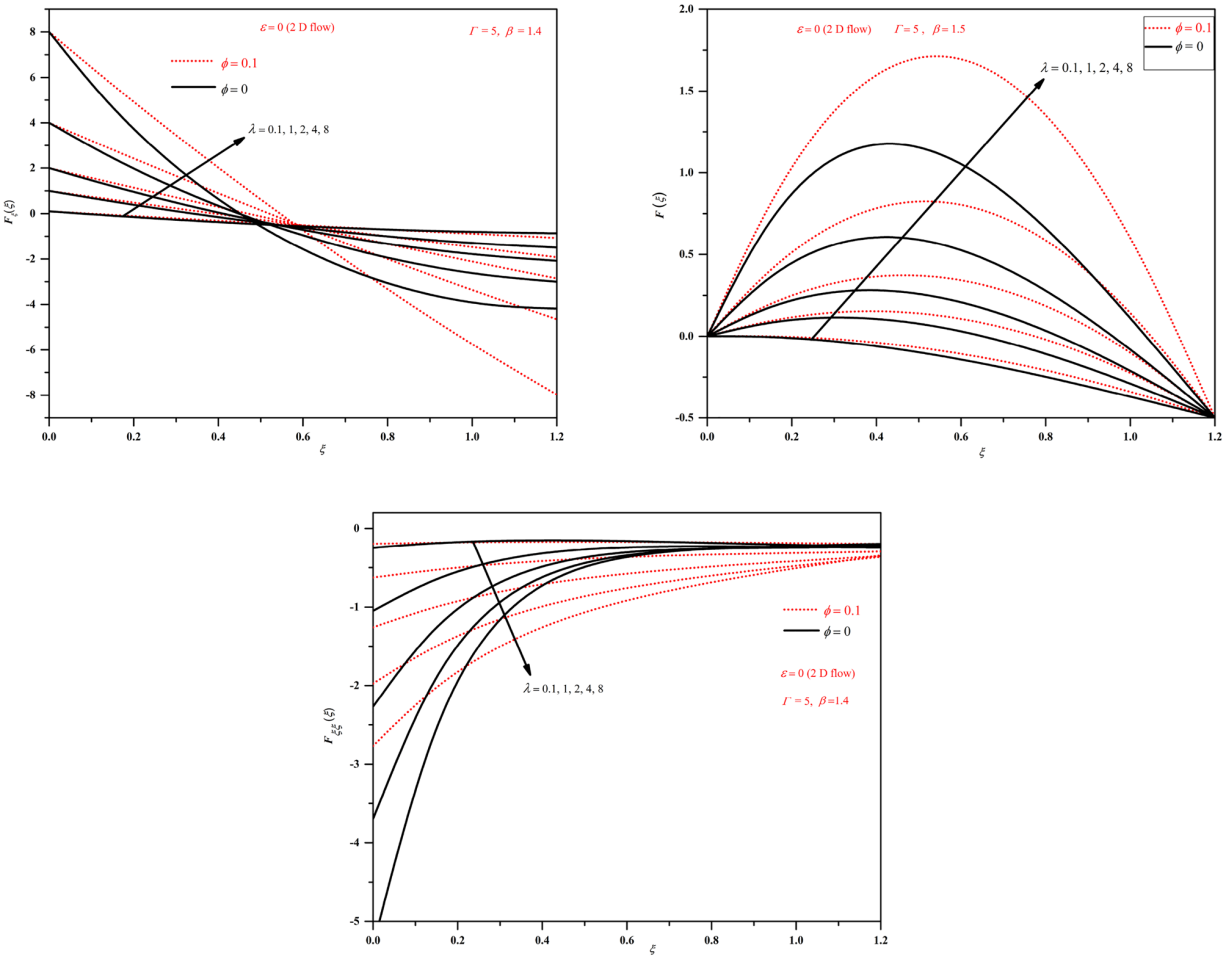
Plots of *F*, $F_{\xi }$ and $F_{\xi \xi }$ by varying $\lambda$ (stretching wall $\lambda >0$) in the two-dimensional case.

**Figure 4:**
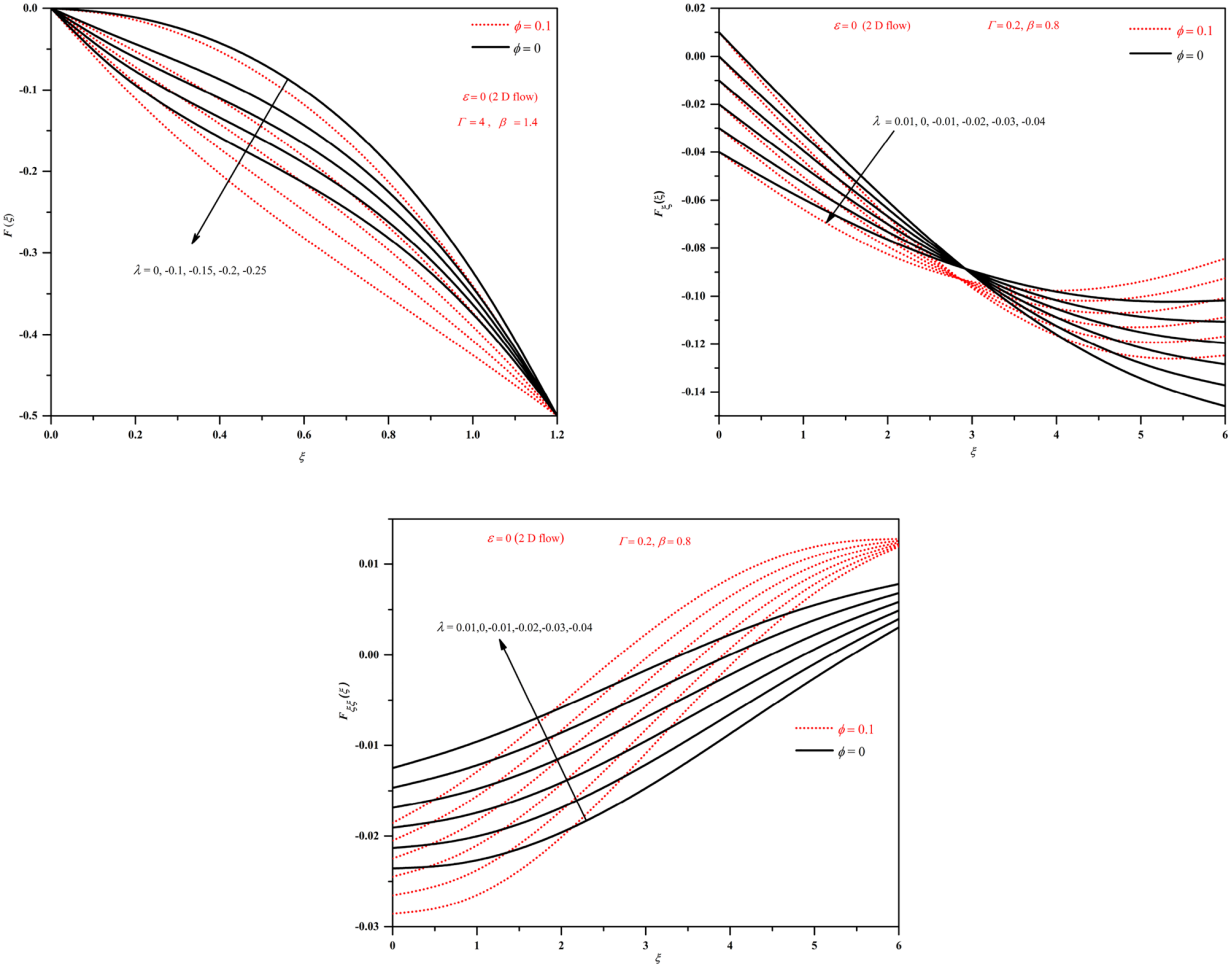
Plots of *F*, $F_{\xi }$ and $F_{\xi \xi }$ by varying $\lambda$ (shrinking wall $\lambda <0$) in the two-dimensional case.

**Figure 5:**
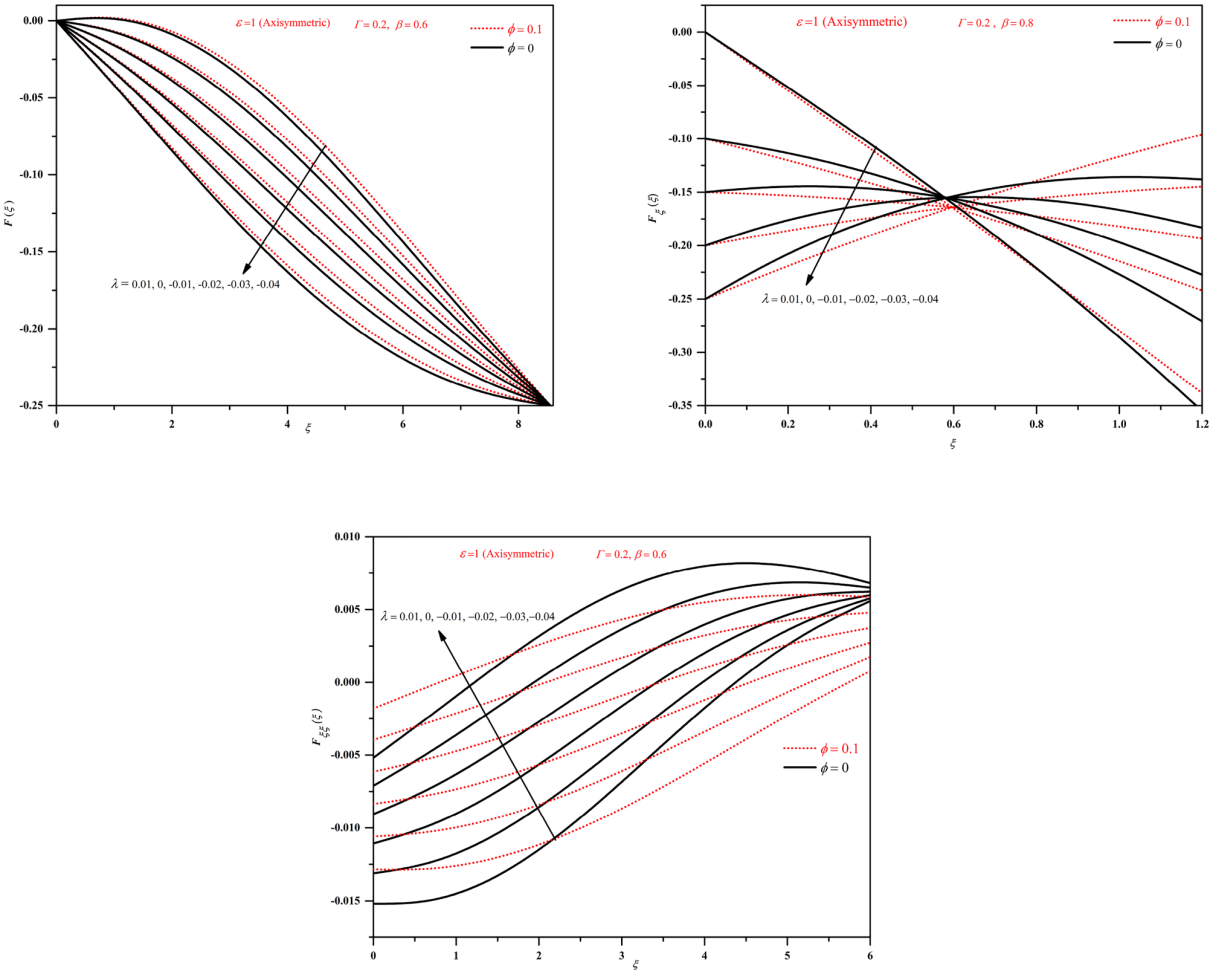
Plots of *F*, $F_{\xi }$ and $F_{\xi \xi }$ by varying $\lambda$ (shrinking wall $\lambda <0$) in the axisymmetric case.

The results in the present study are analysed by considering the nanofluid in which base fluid is water and aluminium oxide nanoparticles. The solutions for various wall moving parameter $\lambda$ are graphically presented in Figs. 2, 3, 4, 5. Figures 2 and 3 show the plots of *F*, $F_{\xi }$ and $F_{\xi \xi }$ for stretching wall condition ($\lambda >0$) with base fluid and with nanofluids for axisymmetric and two-dimensional cases. The shrinking wall condition is investigated on Fig. 4. It can be noticed that *F* and $F_\xi$ shows increased magnitude but $F_{\xi \xi }$ shows decreased magnitude with nanofluids when compared to conventional fluids. We can observe that for $\lambda >0$, stretching wall condition velocity plots show increasing nature with increasing the separation from the wall and as the value of $\lambda$ increases velocity becomes higher. The plots of $F_{\xi \xi }$ shows monotonically decreasing behaviour with increasing $\lambda$ for both two-dimensional and axisymmetric cases. We can notice the higher magnitudes of *F*, $F_{\xi }$ and $F_{\xi \xi }$ for nanofluids.

From Figs. 4 and 5, we can observe that for $\lambda <0$, shrinking wall condition both *F* and $F_{\xi }$ shows decreasing nature but $F_{\xi \xi }$ shows increasing behaviour with decreasing $\lambda$. We can notice the intersecting point in the plots of $F_{\xi }$ near $\xi =0.5$. The behaviour is similar for both two-dimensional and axisymmetric cases and only differs with magnitude as can be observed from the figures. Here also we can notice the higher magnitudes of *F*, $F_{\xi }$ and $F_{\xi \xi }$ for nanofluids.

**Figure 6:**
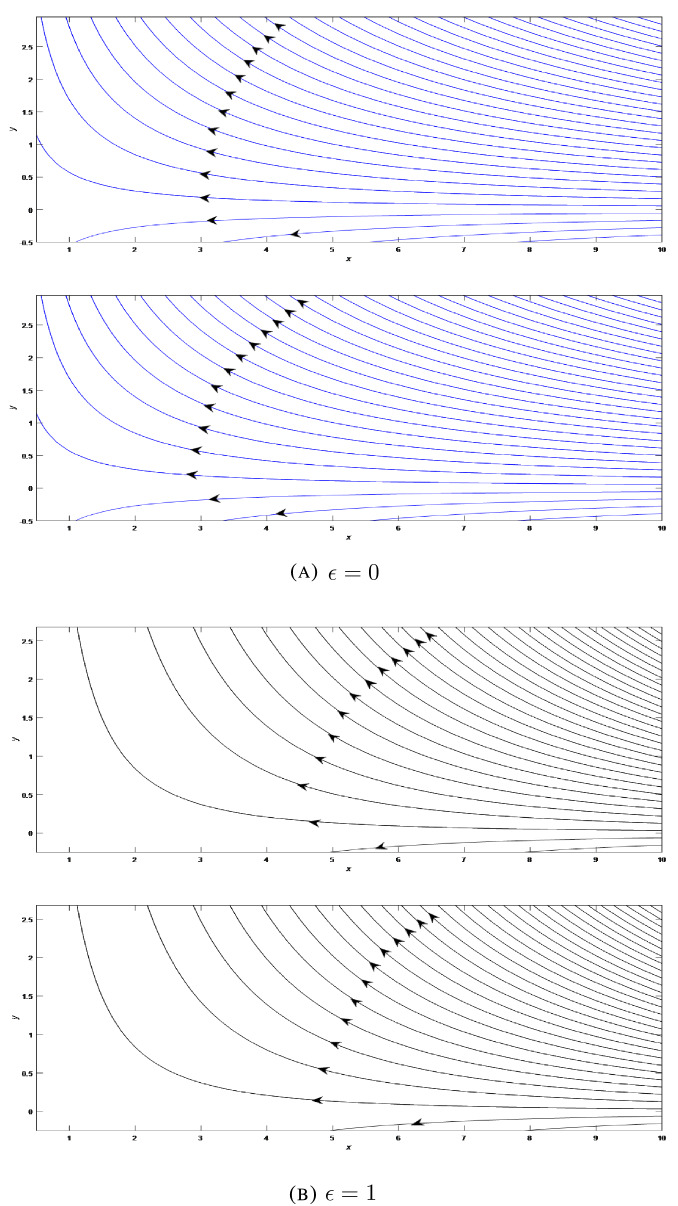
Streamlines of the film flow for varying time steps $t=1$ and $t=5$ for two-dimensional and axisymmetric stretching ($\lambda =2$) flow patterns.

**Figure 7:**
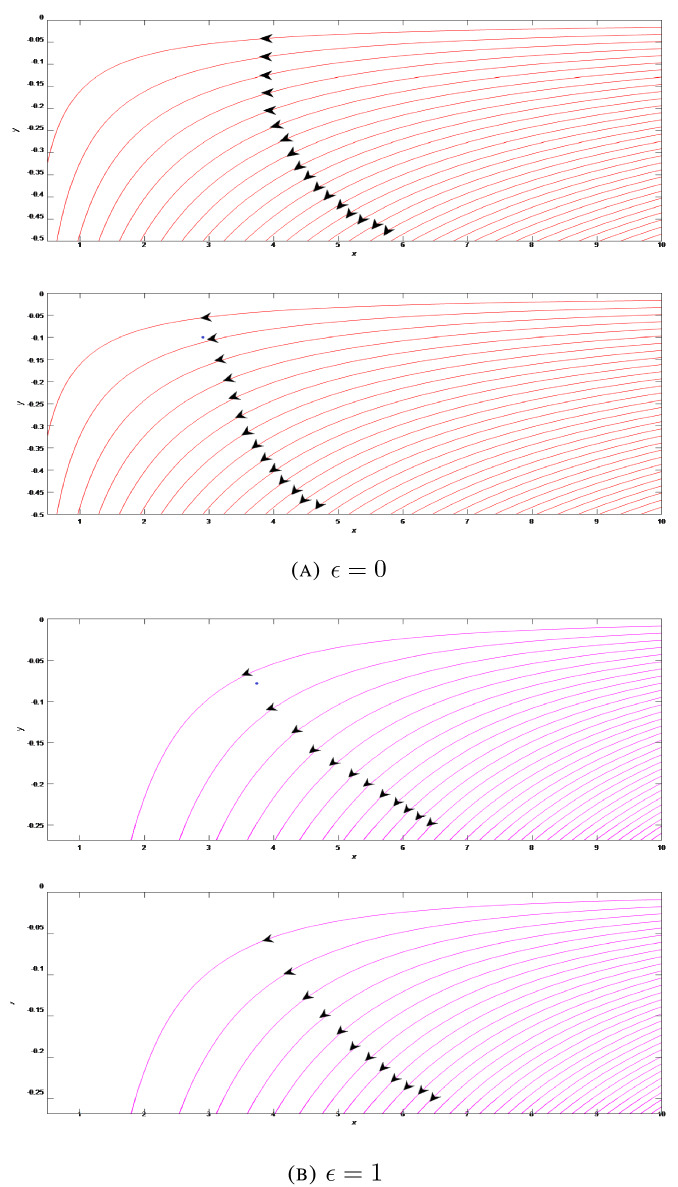
Streamlines of the film flow for varying time steps $t=1$ and $t=5$ for two-dimensional and axisymmetric shrinking ($\lambda =-0.2$) flow patterns.

**Figure 8:**
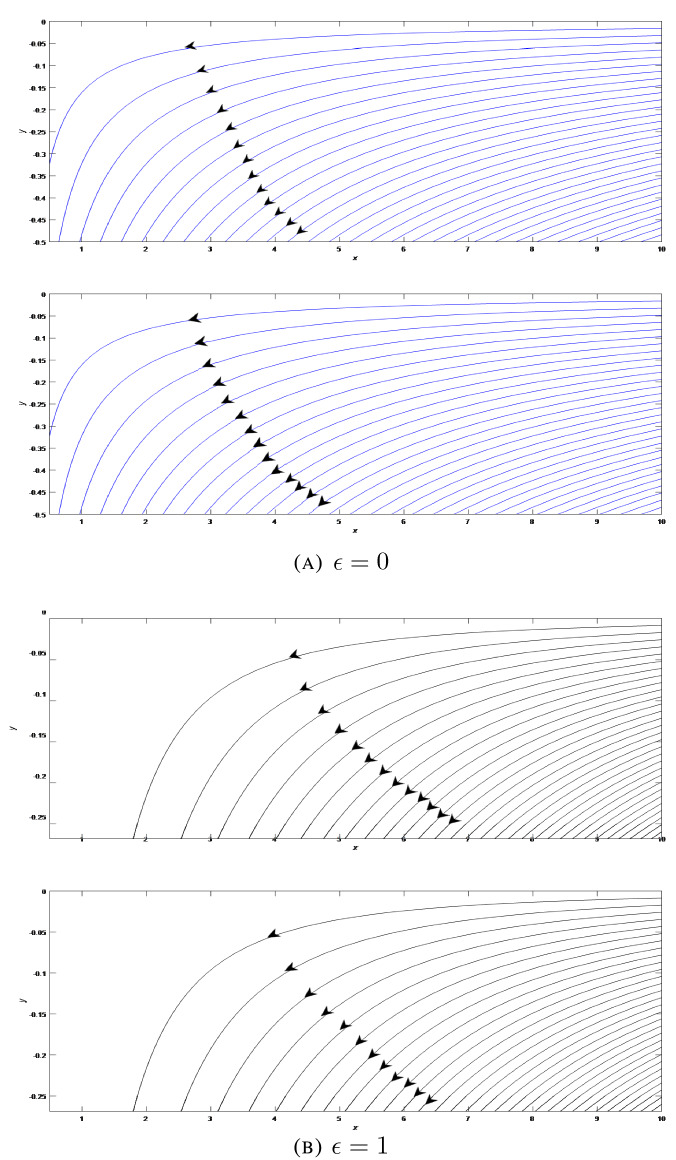
Streamlines of the film flow for varying time steps $t=1$ and $t=5$ for two-dimensional and axisymmetric constant wall ($\lambda =0$) flow patterns.

For further analysis of flow pattern, stream function is defined as $\psi =\sqrt{\frac{1}{t}}x^{1+\epsilon }f\left( \frac{y}{\sqrt{t}}\right)$ and streamlines are plotted as presented in Figs. 6, 7, 8 at different situations with standard units for physical quantities. Kinematic viscosity is presumed to be unit for the sake of discussion of results without loss of generality. Figure 6A presents the flow field in the two-dimensional liquid film with stretching wall ($\lambda >0$) at varying time steps $t=1$ and $t=5$. We can seen that fluid moves to the left and have a streamline with zero velocity which creates two parts in the flow region. In the lower part region fluid starts to move to right side. For axisymmetric case, we can observe the flow has shifted to lower region as shown in Fig. 6B. Flow patterns in this case are almost similar as in the two-dimensional case. We can notice the dense streamlines at higher *x* co-ordinate. For shrinking wall ($\lambda =-0.2$), flow fields are presented in Figs. 7A and B at different time steps $t=1$ and $t=5$ for 2D and axisymmetric cases respectively. Streamlines are oriented in an uniform order and the entire fluid flow in the left direction. The film thickness becomes higher with the growing time. Figures 8A and B show the flow field when the wall is at rest (i.e. $\lambda =0$) at varying time steps $t=1$ and $t=5$ respectively for 2D and axisymmetric flow patterns. In this particular situation, wall is not moving and the film fluid moves to the left.

### Solution behaviour analysis for large $\lambda$

For large values of $\lambda$, there is a particular pattern of $F_{\xi \xi }$. Therefore, to get more intuition for the behaviour of solution for higher scale of *A*, extended analysis was accomplished. By expressing $F=\lambda \phi (\xi )$ and using this into the Eq. (10), we get

15 $$\begin{aligned} \phi _{\xi \xi \xi }+\frac{\beta ^2}{A\left( 1+\frac{1}{\Gamma }\right) }\left[ \lambda (1+\epsilon )\phi \phi _{\xi \xi }-\lambda \phi _{\xi }^2+\left( \frac{\xi }{2}\phi _{\xi \xi }+\phi _{\xi }\right) \right] = 0, \end{aligned}$$

associated with the boundary constraints

16 $$\begin{aligned} \phi (0)=0, \quad \phi '(0)=1, \quad \text{ and } \quad \phi ''(1)=0. \end{aligned}$$

If $\lambda$ is sufficiently larger, the term $\frac{\xi }{2}\phi _{\xi \xi }+\phi _\xi$ becomes negligible when compared to $\lambda (1+\epsilon )\phi \phi _{\xi \xi } -\lambda \phi _\xi ^2$. In order to include or exclude $\phi _{\xi \xi \xi }$, let us simplify Eq. (15) by approximating $\beta$ as $\beta ^2=\sigma ^2\lambda ^\alpha A\left( 1+\frac{1}{\Gamma }\right)$ assuming large $\lambda$ and $\alpha <0$. Using this in Eq. (15), we remains at

17 $$\begin{aligned} \phi _{\xi \xi \xi }+\sigma ^2 \lambda ^{\alpha +1}[(1+\epsilon )\phi \phi _{\xi \xi }-\phi _\xi ^2]=0, \end{aligned}$$

subjected to the boundary constraints

18 $$\begin{aligned} \phi (0)=0, \quad \phi _\xi (0)=1, \quad \phi _{\xi \xi }(1)=0 \quad \text {and} \quad \phi (1)=0. \end{aligned}$$

Based on the value of $\alpha$, Eq. (17) can be reduced into various equations. If $-1<\alpha <0$, for higher scale of $\lambda$, Eq. (17) takes the form

19 $$\begin{aligned} (1+\epsilon )\phi \phi _{\xi \xi }-\phi _\xi ^2=0. \end{aligned}$$

The general solution for Eq. (19) can be obtained as

20 $$\begin{aligned} \phi (\xi )=\xi C_1+\frac{\xi ^2C_1^2}{4C_2}+C_2. \end{aligned}$$

Any two of the boundary conditions (18) are not satisfied by this solution. Hence, $-1<\alpha <0$ is not an appropriate assumption. If $\alpha <-1$, Eq. (17) becomes

21 $$\begin{aligned} \phi _{\xi \xi \xi }=0. \end{aligned}$$

We get parabolic function as the general solution of this given by

22 $$\begin{aligned} \phi (\xi )=C_1\xi ^2+\xi C_2+C_3, \end{aligned}$$

which cannot satisfies the either boundary conditions. An acceptable option is by presuming $\alpha =-1$ with Eq. (17) becomes

23 $$\begin{aligned} \phi _{\xi \xi \xi }+\sigma ^2 [(1+\epsilon )\phi \phi _{\xi \xi }-\phi _{\xi }^2]=0. \end{aligned}$$

## Concluding remarks

The flow of Casson nanoliquid film past an unsteadily moving wall with a specifically described surface velocity has been examined. The regulating Navier–Stokes expressions are modified into a similarity ODE with predefined velocity functions. The resulting similarity expressions are then tackled numerically. Various velocity and shear stress characteristics are witnessed in the two different flow directions whereas the velocity exhibits monotonic deviation having no points of zero-crossing. However, the shear stress exhibits non-monotonic nature at many of the constraints. Flow field shows that for stretching wall, fluid moves to the left and have a streamline with zero velocity which creates two parts in the flow region. In the lower part region fluid starts to move to right side. For axisymmetric case, we can observe the flow has shifted to lower region. For shrinking wall, Streamlines are oriented in the same order and the entire fluid flow in the left direction.

## Data Availability

All data generated or analysed during this study are included in this published article [and its supplementary information files].

## List of symbols

*g*: Gravity acceleration ($$\text {ms}^{-2}$$)
*h*(*t*): Thickness of the fluid film ($\text {m}$)
*t*: Time ($$\text {s}$$)
*p*: Fluid pressure ($$\text {Pa}$$)
$p_0$: Ambient gas pressure (Pa)
*u*: Velocity along *x* direction ($\text {ms}^{-1}$)
$U_w$: Surface moving velocity ($\text {ms}^{-1}$)
*v*: Velocity along *y* direction ($\text {ms}^{-1}$)
*x*, *y*: Coordinate axes

## Greek letters

$$\rho$$: Fluid density ($$\text {kgm}^{-3}$$)
$\beta$: Dimensionless film thickness
$$\psi$$: Stream function
$\Gamma$: Casson fluid parameter
$$\nu$$: Kinematic viscosity ($$\text {Pas}^{-1}$$)
$\epsilon$: Flow configuration parameter
$\lambda$: Wall moving parameter
$$\eta$$: Similarity variable
$\phi$: Nanoparticle volume fraction
